# A Treatise on Diseases of the Nervous System

**Published:** 1822-09-01

**Authors:** 


*A Treatise on Diseases of the Nervous System. Part the First, Convulsive and Maniacal Affections.* By A. C. Prichard, M. D. &c. Octavo, pp. 425. London, 1822.

[Second Analytical Article.]

### MANIA.

Numerous have been the terms, taken almost entirely from the material world, which have been invented to designate that state of mind which is opposed to sanity. The Greeks called it mania, probably from their verb mainomai, *I rage*;—the Romans, delirium, from de lira, *out of the track*;—the French, derangement, *out of rank or order*;—it has also been termed insanity, madness, lunacy, and other appellations, some of the derivations of which are obvious, and others not worth investigating. The *definitions* of the disease have been still more numerous than the names thereof—some of them curious enough. Thus Mead conjectures—"that this disease consists entirely in the strength of the imagination;"—Cullen that "it consists in such false conceptions of the relations of things as lead to irrational emotions or actions;"—Ferriar conceives *false perception*, and consequently confusion of ideas to be a leading phenomenon in insanity;—Haslam (in his first edition) characterised insanity as "an incorrect association of familiar ideas, independent of the prejudices of education, always accompanied with implicit belief, and generally with either violent or depressing passions;" but in the second edition, he gives up the attempt at a definition at all. Halloran and Cox have also declined a definition of the disease. Esquirol has defined mania as "general, and chronic delirium, without fever, and with an excitation of the vital forces." Dr. Prichard, in the work before us, considers insanity as "chiefly distinguished by a general incoherence of thought—the ideas appear scarcely to follow any connected course—the attention passes in a hurried manner from one assemblage to another." But the fact is, that, as in most of the purely corporeal diseases, the various shades, the wide range, the mutable character of insanity will not be coerced into a pithy definition.

Dr. John Monro has given a curious and spirited sketch of the prelusive phenomena which often announce insanity. The person will exhibit what is called high spirits—shall take wine freely, though previously abstemious—from being reserved and modest, he shall talk boldly and obscenely—sit up late, sleep little, and start from bed early—every thing he says or does betraying violent agitation of mind. Yet in the midst of all this hurry he will not misplace a word, or give the least reason for any one to think he *imagines* things to exist that really do not, or that they appear to him different from what they do to other people. Esquirol draws a still more striking picture of the onset of insanity. "What a change," says he, "has been effected in that man, who, only yesterday, was mersed in the most profound meditations, submitting to his calculations the laws of the universe, and balancing, in the vastness of his conceptions, the destinies of empires—whose wisdom was capable of opening new sources of prosperity to his country—whose genius was enriching the arts with the most exquisite chefs d'œuvre! All at once, mistaking every thing around him, and even himself, his mind is transformed to a chaos—his dispositions become perverted, and he wishes to upset and destroy all things. He appears at war with all the world—hates all which he before loved—and looks like the genius of evil amusing himself with the confusion, disorder, and destruction which he scatters around him! That woman, the very image of candour, virtue, and modesty—whose tongue uttered only the most mild expressions and generous sentiments—who was a dutiful daughter, an affectionate wife, a tender mother, loses the empire of reason. Her bashfulness is changed into audacity—her gentleness into ferocity—and from her lips comes nothing but execrations, blasphemy, or obscenity. She no longer regards the laws either of decency or humanity—she exposes herself naked to strangers—and, in her blind delirium, threatens her parents, strikes her husband, or strangles her children, if recovery or death do not terminate the hallucination! To this storm, deplorable as it is, succeeds a calm ten thousand times more afflicting. The maniac falls into a state of careless apathy; he no longer evinces the contention of mind within him—utters no threats against those around him—all recollection of the past is obliterated —his intellectual faculties sink into *dementia*, the tomb of human reason—he becomes an object of pity or disgust to the bye-standers, who scarcely recognize him as *a man*—he drags out the remainder of a *material* life, without desires or regrets—and sinks silently into the grave!"*

There are instances, as we before stated, where insanity makes its approach gradually—a certain waywardness or singularity of character is observed for some time, perhaps for years, before the individual is set down by his friends as a maniac. But in general the disease breaks out suddenly —the manners of the patient become unusually impetuous— his conversation hurried—his mind full of projects, which he pursues with restless activity. He betrays the absolute derangement of his intellect by announcing some false and absurd impression, or by acting upon it. When his attempt is resisted, or when by accident he reveals the motive of it, his condition is rendered evident, and restraint becomes necessary.

At the first attack there is generally a disordered state of the whole, or a part of the system, as febrile excitement, disturbance of the sensorial functions, constipation of the bowels, want of sleep, impaired appetite, flushing of the face, redness of the eyes, contraction of the pupils, pain in the head with throbbing of the arteries leading to it, sometimes giddiness and confusion of sight. Those who have had repeated attacks are occasionally sensible of the return of the malady. Some have described the attack as highly delightful. A patient of Dr. Willis's assured the doctor that he always expected, with impatience, the accession of the paroxysms, since he enjoyed, during their presence, a high degree of pleasure. They lasted ten or twelve hours, during which every thing appeared easy to him. His memory acquired, all at once, a singular degree of perfection—long passages of Latin authors occurred to his mind, and he could write in verse as easy as in prose. He found himself cunning, malicious, and fertile in all kinds of expedients. Some have described a sense of working in the head, and also in the intestines, like a fermentation. Others have observed that they

* Esquirol on Mania; Dict. des Sciences Medicales, Vol. xxx. p. 437.

did not seem to possess their natural feelings—and all agree that, they become confused, from the sudden and rapid intrusion of unconnected thoughts.

Dr. Haslam remarks, that on the approach of mania, the patients become uneasy and incapable of confining their attention, neglect their employment, get little sleep, become loquacious and disposed to harrangue, decide promptly and positively upon every subject—then they begin to divest themselves of all restraint, declare freely their opinions of their acquaintances, express with fervency and extravagance their friendships and enmities, become impatient of contradiction, and scorn reproof. Many have all the appearances of inebriation, to which the approach of mania often bears a striking similitude. At length, suspicions creep upon the mind—they seem aware of plots that were never contrived, and detect motives that were never entertained. Last of all, the succession of ideas becomes too rapid for examination—the mind becomes crowded with thoughts, and confusion ensues.

Those under the influence of the depressing passions, exhibit a different train of symptoms. The countenance wears a gloomy and anxious aspect—they are averse from conversation—retire from the company of their former associates—seclude themselves in obscure places, or lie in bed the greater part of the day. They next become fearful, and conceive a thousand fancies—often allude to some immoral act which they have committed, or imagine themselves guilty of some crime which they have never perpetrated. Frequently they become desperate, and attempt, by suicide, to free themselves from an existence which has become an afflicting and hateful incumbrance.

The mental characteristics of this disorder involve all those aberrations from sound intellect which render man a useless, and often a dangerous member of society. A degree of cunning, not always to be divined even by those most acquainted with the insane, constitutes a leading feature in mental derangement. Whether they have meditated destruction to themselves, or mischief to others, the accomplishment of the deed is often the only notice of the intention. Dr. Prichard, in shewing the defective definitions which have been given of insanity, combats the idea of Cullen that, false perceptions or recollections, produce *disproportionate emotions* in the insane.

"The emotions of a lunatic are indeed often vehement, and are forcibly expressed; but it may very well be doubted whether they are out of just proportion to the mental impressions from which they arise, or are in reality more vivid than those which many sane persons, of susceptible temperament, would experience, were they actually placed in the precise circumstances with which the imagination of the lunatic environs him. A madman will often fancy himself a king, and then he will utter expressions of violent indignation if he is not treated with all the respect and obedience to which his elevated station entitles him: but I believe there is many an autocrat who would be just as grievously affronted, if his royal honours were treated with as much freedom and contempt as the poor lunatic is fated to experience. Give the latter the obeisance which he fancies to be his due, and he will be infinitely gracious and condescending." P. 119.

Dr. Prichard has come to the conclusion that, in madness, it is not the *reasoning or judging* faculty which is involved—nor does he think there is any primary derangement of the emotions or passions, which, he believes, are always in proportion to the impressions from which they take their rise. Neither can erroneous or impaired *sensation* be the cause of insanity, for in such cases there is no correspondent error of perception, the false impression on the organ of sense being immediately corrected by the mind. Nor can madness, our author observes, be said to consist chiefly and essentially in error or defect of *perception*—since lunatics in general have very acute perceptions, and distinguish very clearly and correctly the persons and objects that surround them. If then we exclude from the idea of insanity, the intellectual phenomena of sensation, perception, and the reasoning or judging faculty, what are we to regard as the essential circumstances of the disease?

"It will be found, if I am not mistaken, that the faculties of the mind, to which we must direct our chief attention in investigating the nature of madness, are memory and imagination, or reverie; and, in fact, that the habit which characterizes a lunatic is that of confounding the results of these two mental operations, and mistaking the ideas of reverie for the impressions of memory and reflection." 123.

We cannot detail the arguments which our author brings forward in support of this position; but he concludes, that the reveries of the madman—"even the most idle and fantastical of them, produce upon his mind the same effect, and leave exactly the same impression, as in a sane person takes place through the medium of actual perception, or of the assemblage and combination of the ideas of perception, by the active exertion of memory and reflection. To sum up this account in a few words, the character of madness seems to consist in the circumstance that *the impressions of reverie are so modified by the disease as to be no longer distinguishable from those of attentive and active reflection*."

Those forms of madness wherein the patient fancies that he perceives things or persons present, when they have no existence, appear to militate against our author's belief, that there is no *false perception.* But he attempts to explain the difficulty, by supposing that, when the maniacal hallucination becomes exalted to a very intense degree, it represents unreal objects as actually present—that is, certain phantasms, the creations of reverie or imagination, are presented to the mind in such vivid colours, as to produce a similar effect to that of actual perceptions—the patient, in other respects making no mistake with regard to place, time, or surrounding objects. Thus, Dr. P. has seen a lunatic under this form of the disease, walk up and down a street, sufficiently alive to external objects to avoid falling in the way of horses and carriages, or running against foot passengers, but yet, so intent upon the scene presented by his reverie, as to be busily employed in issuing commands to troops of soldiers, of which he imagined himself to be the general, and directing them to enfilade and perform a variety of evolutions. All this he performed with a voice and gesture which were perfectly natural, and consistent with reality. To this class of madness belongs, Dr. P. thinks, what have been termed idolo-mania, or dæmonomania, in which the lunatic fancies that he sees and holds conversation with imaginary beings; the conception of the mind being so vivid and intense as to withdraw the attention from all surrounding objects, but without actual inability to form a correct conception, if once the attention be diverted from the subject of hallucination.

After all, it must be confessed, that we know not by what physical cause those affections of the mind are produced which are termed impressions of memory—nor do we know in what respect these operations differ from those peculiar to reverie—we are not, therefore, prepared to explain how the former can be converted into the latter. But let us come to more tangible points.

There are certain bodily appearances which are said to indicate insanity, and to be distinguishable by those conversant with the insane. These are, a peculiar cast of countenance not to be represented by graphic description—a quick, oftentimes protruded and glistening eye—coldness of the hands and feet—capability of sustaining cold with comparative impunity (but this is denied by Pinel and some others)—obstinate torpor of the bowels—tinnitus aurium, and other aural affections—relaxation of the scalp, (observed by Dr. Haslam,) especially after a raving paroxysm of some continuance.

*Pathology of the Brain in Mania.* Dr. Prichard observes that, most of the remarks which he offered on the subject of epilepsy,* are applicable to mania. It has been a common complaint, that little has been found in the brains of maniacs to elucidate the disease—physicians, he thinks, having looked too much for some peculiar phenomenon distinctive of this particular malady; whereas, if he be right in the observations which he has made on nervous disorders, "all that can be expected to be found, are the common vestiges of increased vascular fulness, whether inflammatory or congestive." To the effects of increased vascular action, he supposes we may refer all the phenomena usually found in the brains of maniacs, without excepting that preternatural hardness, described by authors, or even the bony depositions so frequently seen about the dura mater. In confirmation of this statement, Dr. Prichard quotes a passage from Pinel, in which that experienced physician remarks that, "he had attended at thirty six dissections in the hospital Bicêtre, and he never met with any other appearances within the cavity of the cranium than are observable on opening the bodies of persons who have died of *apoplexy*, *epilepsy*, *nervous fevers*, and *convulsions*." As a counterbalance to this, however, we may refer our readers to M. Esquirol's writings, and particularly to page 257 of the first volume of this series of the Journal, in which it will be seen that—"in numerous and accurate dissections of the insane, no alteration whatever from the healthy structure could be discovered."† Esquirol, indeed, admits that the disease is always occasioned by some corporeal lesion, whether of structure or function; but, he thinks, that this lesion is not exclusively in the brain, the vital powers or intellectual operations of which, become affected secondarily in many cases.

Chiarugi in Italy, Greding in Germany, and Haslam and others in this country, have detected diseases of the brain or its membranes in the insane; but there may exist many alterations, perhaps even of structure, in so delicate an organ as the brain, too minute for the eye to observe, and not to be demonstrated by the scalpel. The membranes of the brain are as frequently found altered, as the cerebral substance. The tunica arachnoidea becomes thickened, and rendered more or less opake—the pia mater more or less inflamed, turgid with blood, and not unfrequently with an extravasated blotch on this tunic. Effusion of a watery fluid between the membranes is a very common occurrence, as well as into the ventricles.

---

* See page 129 *et seq.* of our last number.

† Med. Chir. Rev. (Analytical Series) Vol. I. p. 258.

*Etiology.* Dr. Prichard prefaces the subject of *uterine* mania with some judicious observations on the pathology of nervous affections in general, which are connected with the state of the uterine functions. He does not seem to adopt the usual division of maniacal causes, into moral and physical. He probably looks upon the *physical* causes as the only efficient ones, and without which, the disease would not exist. We believe, indeed, that moral causes only act in producing those physical disorders that, in turn, react upon the mind.

Esquirol remarks that, of all the causes of mania, in females, irregularities of the menstrual secretion are the most common. The next is the state of lactation, whether the milk fails to come to the breats after parturition, or become suppressed in the course of suckling, or the woman neglects the proper management in weaning. Insolation, or exposure to great heat, is set down by M. Esquirol as a frequent physical cause of insanity. Repressed eruptive diseases, that have been long established, occasionally excite the maniacal state, particularly in the period between 35 and 45 years of age. It is in such cases, that issues or other drains from the system, are so useful. Epilepsy is a frequent cause of the disease. Of 400 epileptic patients in the Salpetriere, fifty and upwards are maniacs—and those of the most violent kind; but the paroxysms are generally of short duration—sometimes only a few hours—sometimes three, four, or eight days. These paroxysms of mania rarely *precede*, but generally *succeed*, the epileptic attack.

Among the physical causes of insanity we ought to have alluded to *hereditary* disposition, which performs so prominent a part in the etiology of mania. M. Esquirol justly regards the moral, as far more numerous than the physical, causes of insanity—especially in the female sex. These moral causes are also much more frequently in action among the higher, than among the lower orders of society. In the former class, the intellectual faculties are more exercised and developed—the passions more excited, and more energetic. More dependent on the caprices of fortune and of men, the rich are far more exposed to the chagrins resulting from wounded self-love, and vicissitudes of circumstance, than the poor. In the upper classes of the female sex, the passion of love is continually interrupted and crossed in a thousand different ways, and thus becomes a prolific source of insanity. Esquirol remarks, and we think the remark just, that it is rare to see either moral or physical causes *singly* produce insanity—they are generally combined, or one is the effect of the other. Thus, a fright will suppress the menses, and this suppression will cause madness, which will disappear when the catamenia are restored.

But to return to Dr. Prichard's work, sect. 5 of chap. 5. Maniacal affections, he observes, are connected in a variety of modes with the uterine functions. Sudden suppression of the catamenia from cold, over-excitement, or any powerful mental emotion which disturbs the system, is occasionally the prelude to a maniacal attack, which is generally of short duration, subsiding when the catamenia are restored. Sometimes, however, the effect will remain after the cause is removed. We know that many, indeed we might say, *most* women display greater or less degrees of excitement at the periods of menstruation, even when these are regular. In some, these amount only to a depression of spirits or irritability of temper—in others, to hysterical symptoms—in others again, maniacal impressions take hold of the mind. When therefore we find aberrations of intellect conjoined with great irregularity of menstruation in young women, we may, with much probability conclude, the latter to be the cause of the former. In respect to the nature and treatment of uterine mania, our author supposes it will be readily allowed that the theory which applies to uterine epilepsy will apply here.* In a large majority of the cases of uterine epilepsy and uterine mania, the women were of strongly marked sanguine temperaments, an additional fact in favour of the analogy between the two classes of disease. But independently of all analogy, the phenomena of the disease and the effects of remedies sufficiently point out the course to be pursued in uterine mania.

"One observation which I have made respecting the treatment of these disorders is the following. In uterine mania, more may be expected from the effect of stimulating emmenagogues than in analogous instances of epilepsy; and frequent and copious bleedings are not, in general, so necessary or so safe in the former disease as in the latter." 201.

Dr. P. considers the tincture of melampodium and the oil of turpentine to be the most efficacious emmenagogues in cases of uterine mania—the former in doses of thirty to sixty drops thrice a day. Our author has so often prescribed this medicine in amenorrhœa, and has so frequently seen the catamenial flow ensue, that he is convinced it possesses some specific power in promoting this discharge.

"But the oil of turpentine is generally more efficacious. This medicine is a most powerful and diffusible stimulant; it acts on several of the secretions, particularly on that of the kidney, and often occasions even hæmaturia. There is no other substance more likely, from its known properties, to exert an influence on the secre-

---

* See uterine epilepsy, p. 182 *et seq.* of last number.

tive action of the uterus. With this view I have prescribed it in the form of an emulsion, each dose containing from half a dram to a dram of the rectified oil, to be taken three times in a day. Sometimes I have preferred to give two drams of the oil at night, or a double quantity during the day, together with some brisk purgative. Clysters of ol. ricini and ol. terebinth. of each an ounce, are often successful in bringing the same result. The use of the warm bath should be ordered at the same time." 203.

Of other stimulants, as the balsams and the lytta, our author has not had sufficient experience. Chalybeates he has seldom or never prescribed, from a conviction of their impropriety in such cases. In other respects the treatment of uterine mania must be conducted on the same principles as uterine epilepsy.* Four cases are related in illustration of maniacal disease connected with dysmenorrhœa, or with suppression of the catamenia. Of these we shall notice one or two instances.

*Case* I. Rebecca James, admitted June 5th, 1820, of sanguine temperament, and aged 30 years. She had been in hospital for the same complaint about nine months before. She talks incessantly—is sometimes very boisterous—extremely irritable—subject to sudden flushings of the face. When the excitement is over she remains sullen, or cries and laughs alternately. Bowels are torpid—pulse rapid—catamenia irregular, being sometimes wanting, and always scanty when they do appear. Twenty-four ounces of blood from the back of the neck by cupping—a blister—an emetic—a purgative. Next day, there being little alteration, she was bled from the arm, and had powders of antimony, opium, and jalap. Ten days after entering the hospital, the cold shower bath was ordered. 21st July, very noisy—"sent to the pens." 27th, in a state of collapse—cries and laughs, or is sullen—" pil. confect. hyd. cum aloe." Aug. 19th, convalescent, and soon afterwards discharged apparently well. She returned, as an out patient, in March 1821, alarmed at the idea of a relapse, having some premonitory symptoms, and a delay of the catamenia. She was bled—took the turpentine emulsion, and tincture of black helebore. The catamenia soon followed. She was completely relieved. A relapse of mania occurred in June 1821, but was removed by evacuants.

*Case* II. The history of this case we shall give in our author's own words.

---

* See page 134 *et seq.* of No. IX.

"Ann Marsh, aged 35, admitted May 25, 1816. An unmarried woman, of middle stature, full robust habit, short neck; face generally flushed; sanguine complexion, viz. light blue eyes, light brown hair. She complains of being extremely nervous: says she has been subject to giddiness from her infancy. She has been for some years a cook, and fancies that her present complaints were brought on by standing near the fire.

"Her complaint is not hereditary; it has been gradually coming on for some months. She would frequently start from bed and walk about the room. But it is within the last nine days that the disorder has assumed its present form.

"At present her countenance is extremely wild; the pupils of her eyes are contracted, her bowels costive, and her breath offensive. She is very unmanageable, and at times utters a loud dismal shriek, which is always the forerunner of a violent struggle; she kicks and tears every thing near her until she gets into a profuse perspiration.

"*State of the natural functions.*—Bowels costive; breath offensive; appetite keen; catamenia irregular; whenever they are in the least checked, (that is, when they fail to appear at the due time,) the disorder flies to the head: latterly, if any thing vexes her, her head is affected. She says, that if she can cry well her head will not be so much distressed. When at the best, she talks incessantly. She is very irritable, and cannot bear stimuli of any kind." 198.

The head was shaved and blistered—the blister to be kept open—purged freely—saline antimonials every four hours. 5th June, the febrile symptoms had abated. The medicines continued. 12th June, complained of excruciating pain in the head. Twenty ounces of blood from the temporal artery. From this time she gradually recovered. In March 1819, she had a relapse; being suddenly seized with darting pain across the forehead—flushed countenance—suffused eyes—hot skin—quick pulse, &c. Head shaved—sixteen ounces of blood from the temporal artery—cathartic draught—low diet. By these means she was restored to health of body and mind. In August following she had another attack, and was relieved by the same means. Since that time she has continued well, with the exception of some irritability of temper, and a propensity to talkativeness.

*Case* III. This was the mother of fifteen children. During pregnancy of her last child, she complained of what she termed rheumatism in the head, and was never regular in her catamenial periods after parturition. They have appeared at long intervals, and then scanty. She has been in a state of melancholy, and disposed to suicide. Mercurial alteratives and bitters—return of catamenia—much improvement in mind and body—by continuation of the medicines she recovered.

The 4th case related by Dr. Prichard was one of puerperal mania, preceded, however, by threatenings of mental derangement. Evacuations by bleeding and purging gradually restored the patient to sanity of mind and body, though she was still subject to returns of excitement at the catamenial terms. Some months after recovery she became suddenly frantic, in consequence of a fright, and fell into a state of raving and incoherent insanity. In this condition she refused all sustenance, passed her evacuations involuntarily, and sunk comatose. Permission was not obtained to open the body.

Dr. Prichard passes over the subject of puerperal mania with very little notice; and only gives two or three cases in elucidation. With Dr. Ferriar, he is inclined to consider puerperal mania as a case of conversion. "During gestation," says Dr. Ferriar, "and after delivery, when the milk begins to flow, the balance of the circulation is so greatly disturbed as to be liable to much disorder from the application of any exciting cause. If, therefore, cold, affecting the head, violent noises, want of sleep, or uneasy thoughts, distress a puerperal patient, before the determination of blood to the breasts is regularly made, the impetus may be readily converted to the head, and produce either hysteria or insanity, according to its force, and the nature of the occasional cause."*

We refer our readers for further particulars to M. Esquirol's paper on puerperal mania in this volume.

The 8th Section of this chapter of Dr. Prichard's work, contains some observations on maniacal affections as they occur about the cessation of the menses. At this period, we all know, the female constitution is particularly obnoxious to irregular distributions of blood, and the morbid effects resulting thence. Every old woman, indeed, is familiar with this part of pathology, and may perhaps give as good an *explanation* of the phenomenon as any physiologist. The predisposition to insanity, at this period of life, (for our author thinks that no individual can become the victim of insanity, unless he has a constitutional tendency to it, "derived from his forefathers or springing up anew in his original conformation") is greatly promoted by a sedentary life, indulgence in stimulating regimen, and inattention to the bowels. And as extremes approximate in their effects, a a similar consequence often results from an opposite mode of life. Women of the lower orders, who labour hard and

* Med. Hist and Reflect. vol. ii.

frequently beyond their strength, especially in hot weather, (circumstances which stimulate the vascular system,) are liable to this disorder—and contemplative habits, superstitious impressions, the effects of false representations of religion—all mental habits which render the impressions of reverei vivid, and withdraw the attention from objects of sensation and perception, tend to foster this disposition; yet all these might exist for many years, without producing a morbid effect, if the habitual resource, by which the constitution had relieved itself of a burthensome plethora, or of accumulating irritability, had not ceased to avail itself. We shall here abstract a case or two in illustration.

*Case* IV. Sarah A.———, ætat. 58. She is a fat short woman, of melancholic complexion, and strongly marked features. Her brother and sister had died maniacal, the former having committed suicide.

" She has, for several years, led a very solitary inactive life; kept a huckster's shop; used to read a great deal, and spend all her time alone. It seems that she was in the habit of indulging herself in eating and drinking.

" Pulse full. Bowels regular at the present time. Appetite good; rather voracious. She appears always cheeful, and struts about the ward.

" Pil. Cath. Mist. Cath. Low diet.

" Oct. 22. No material alteration. Seems to have been relieved by the shower bath, which she has used. Sleeps little; is garrulous. Pulse natural. Begs for a full diet." 210.

Full diet was accordingly allowed, with a cathartic twice a week, and the cold affusion. But the full diet aggravated the complaint, and rendered her more noisy and troublesome. No medicine indeed produced any material alteration, but she was always worse after repletion.

After nearly two years' continuance of the disease, fever came on after taking a full meal of broth. She was bled and purged, and she seemed recovering. She was allowed porter and other stimulants, and four days afterwards expired.

*Dissection.* Calvaria heavy—vessels of the dura mater turgid—strong adhesions between the latter membrane and skull—a large piece of bony substance at the inner margin of the falx, near its origin. Vessels of the pia mater turgid—that membrane thickened and opake in patches—effusion of serous fluid beneath the pia mater—cerebral substance firm and hard—vessels of the medullary texture minutely injected—lateral ventricles distended with fluid—plexus choroides pale—vessels of the cerebellum loaded with blood.

What is usually termed *religious* madness, we have no doubt is generally a *physical* derangement acting on a mind disposed to religious contemplations, but perverting, of course, the emotions that would naturally rise in the mind of a sane and religious person. A case is related by Dr. Prichard at page 213 of his work, which strengthens this opinion.

*Case* V. Anne Howell, aged 53, of dark complexion, melancholic temperament, "an exquisite example of what is termed religious madness," lies in bed—has the most gloomy and dejected aspect—moans and complains in a tone of unvarying despair—sometimes utters the most frightful shrieks and yells, so as to render confinement in the solitary apartment necessary—gives, on interrogation, the most pitiable account of her miseries, which she solemnly avers to be realities, and not the chimeras of the imagination.

> "She believes that she is the object of the eternal wrath of an offended God, on account of her sins. At night she looks out of the window, and sees the gulf of hell yawning to receive her, and myriads of devils in the midst of fire and brimstone. Being told that God is merciful to those who repent of their sins, she replies, that his clemency extends only to those who have a broken and contrite spirit, and that her heart is hardened and dried up within her. She is as fully persuaded that she is eternally damned as she is of her existence." 213.

On investigation it was found that the disease had made its first attack about the period when the catamenia cease; in consequence, it appeared, of hard labour, exposure, and over-exertion, while working in the open air carrying bark in a tanyard. After suddenly stooping to raise a heavy burden, she cried out that she was seized with a severe pain in the back of her head and neck. When taken home and confined to her bed, her senses were confused, and she complained of undefined feelings of distress. Her apprehensions were directed at first to the state of her body; but being desired to pray and read the bible, on opening it she immediately felt that the wrath of God was denounced against her.

Her health was much out of order, particularly the digestive organs—and pulse rather full. When first received into hospital, she underwent topical depletion and purging, in consequence of which, she was so much relieved that her husband had her removed home. She was re-admitted in

November, 1819; but from this time all remedial measures failed to afford any substantial relief. It was remarkable, however, that, at one time, dark-coloured patches appeared on her thighs, and subsequently on different parts of her body—*she then became sane in mind.* She is now (March 1821) much emaciated, with a strong pulsation in the lower part of the abdomen.

The sixth chapter is on the subject of metastasis to the brain, producing mania and epilepsy—the former disease being omitted in our first analytical article.* We touched on the subject of metastasis, however, in such a manner as will render it unnecessary to say much on that point in the present case. It may be remarked that, if mania be produced by metastasis *to* the head, it is also sometimes cured by the supervention of another diseased action in a part, or even in the whole of the system. Dr. Prichard relates three cases of mania cured by attacks of contagious fever during the late epidemics. In St. Peter's Hospital the lunatics are placed in the same wards with patients labouring under contagious fever—in consequence of which, our author had frequent opportunities of witnessing the effects of fever communicated to maniacs. The principles of treatment in these metastatic cases are detailed in our first analysis, page 138 *et seq.*

### ENTERIC MANIA.

In the first volume of our *quarterly* series, No. 2, for October 1818, we gave an account of Dr. Edward Percival's excellent report on the connexion between maniacal affections and disordered states of the abdominal viscera. Dr. Prichard characterises Dr. Percival's paper as a truly graphical description of enteric mania. Dr. Prichard, however, differs in one point from Dr. Percival—he does not consider a depraved state of the intestinal canal as so very general a feature in maniacal affections as Dr. Percival seems to view it. Still Dr. Prichard admits that "this is one of the most frequent forms under which maniacal disorders present themselves to our notice." There is nothing peculiar in the mental phenomena occurring in enteric mania. It most frequently takes place between the periods of 25 and 40 years of age—is often periodical—the first attack being generally after irregularities in the functions of the stomach and bowels. Our author has notes of many cases where the disease occurred immediately after a long voyage, during which the patient had been fed upon salt provisions, and suffered his

---

* See page 138 of this volume.

bowels to become constipated. Irregular diet and the habitual use of ardent spirits are among the most frequent of the previous circumstances.

"Great anxiety of mind, unusual exertions in business, and especially an effort to grasp at a greater variety of objects, or to engage in a greater diversity of pursuits than the mental powers of the individual qualify him for, will often be found to precede the attacks of this disease; the patient having neglected the state of the natural functions, which an unusual excitement of the nervous system had contributed to throw into disorder. A trifling degree of incoherence, a hurry and confusion of thought; sometimes an absurd degree of energy, manifested in the pursuit of some trifling object, is the first symptom which betrays the actual condition of the patient. In an attempt to reason with him, or resist him, he commonly becomes violent: he has often very early a lurking suspicion of his deranged state: at least this would appear to be the case from the frequent and positive assurances he makes to the contrary, even at times when no suspicion has been hinted.*" 247.

The diseased state of the alimentary canal is generally very strongly marked—the gastric, biliary, and intestinal secretions being depraved. The phenomena would lead to the supposition that chronic inflammatory action was seated in the mucous membrane of great part of the alimentary canal.† In this form of the disease constipation seems to be, as it were, the natural character of the complaint. On enquiry we will be told that the patient has passed six or seven days without a motion—and, when cathartics are administered, a large quantity of excrements is discharged, of an unnatural appearance, the fæces being sometimes dark as coffee grounds, or of a dirty green colour, and very fetid. A long-continued torpor occasionally gives place to a diarrhœa, which usually augments the evil—the abdomen becoming more distended than before—flatulence being added to the load of solid contents, which are only partially discharged. The evacuations are generally thin and watery; or contain mucus mixed with vitiated bile, and recent aliment undigested—sharp and transient pains are experienced in various parts of the abdomen—a quantity of wind is discharged, or rattles

---

"* I have sometimes observed a maniac, after saying something extremely absurd, (although I have taken care that nothing in my countenance or manner of conversing betrayed my impression of his insanity,) as if suddenly struck himself with the apparent incongruity of what he had been saying, break off and protest that he was in his right senses."

† We cannot agree with our author on this point. Constipation of the bowels is a phenomenon quite inconsistent with inflammation of the *mucous* membrane.

about in the bowels—at length dysentery supervenes, and often carries off the patient, or reduces him to a state of extreme emaciation.

"The mouth and fauces, if examined, generally present a diseased aspect. The fauces and velum pendulum are red, the vessels injected, covered in patches with mucus. The tongue is often red; sometimes red with white streaks: more generally, especially when there is diarrhœa, covered partially with a brownish fur. The mouth is viscid, and the patient generally spits out a frothy slime in all directions. There is an ardent thirst, and a peculiar fetor of the breath, which indeed extends to the whole person, and would induce a suspicion that the secretions are loaded with absorbed excrementitious matter." 249.

The appetite is irregular—sometimes there is an aversion to all food, so that the patient cannot be induced to take sufficient nourishment for the support of life—in other instances, there is a voracious desire for food, which is greedily devoured, without selection.

The skin is clammy and cold, with often a remarkable coldness of the extremities, resulting from want of energy in the extreme vessels. In some protracted cases there are papular or scaly eruptions—or furunculi appear in various parts of the body, which are disposed to slough, especially in debilitated subjects.

The complexion is often flushed—the eyes wild, glossy, and lachrymal—the tunica conjunctiva frequently injected—the eye intolerant of light—the pupils more or less contracted the urine is scanty and high coloured, containing matter that should pass by the alimentary canal. The pulse is quick and irritable, the carotids beating with more than proportional force. The patient is sleepless, often for many nights in succession—irritable and tremulous.

The progress of this form of insanity is various. Sometimes a diarrhœa affords relief, the disease either continuing in a mitigated form, or recovery takes place—in other instances, the diarrhœa carries off the patient.

"In more protracted cases, the diseased state of the mucous membrane of the intestinal canal gives rise to glandular obstructions in the mesentery: at least it is a fact that disorganizations of this description are often discovered after death. Hectic fever, with great emaciation, follows, and the patient sinks under a general cachexia, or effusion takes place into the abdomen, and he dies dropsical." P. 250.

Such is a sketch of enteric mania, on the *ratio symptomatum* of which, our author has not much to say. That the affection of the brain producing mania, is connected with the dirordered state of the intestinal canal, as an effect with its cause, Dr. Prichard infers from the analogy of other nervous diseases, which are found to be dependent on morbid states of the enteric functions—and from this important fact that, in the particular instance of mania, agents which produce a change in the intestinal canal relieve, or aggravate, or modify the character of the cerebral disorder.

*Treatment of Enteric Mania.* The same general principles laid down for the treatment of enteric epilepsy will apply here*—namely, a restoration of healthy function in the digestive organs, while, as in epilepsy, the secondary or sympathetic disorder, must be controlled if possible.

Although the abstraction of blood has comparatively a small place in the treatment of enteric mania, yet, it cannot, without impunity, be omitted under certain circumstances.

"When the disease commences with symptoms approaching to phrenitis, with raving delirium, a rapid bounding pulse, particularly in the carotids, a flushed countenance, reddened eyes, heated scalp, dry tongue, intense thirst, it would be wrong to omit bleeding either from the arm or jugular vein. It is much safer to attempt to relieve the sanguiferous system in this method than by emetics; to which recourse has often been made, as if they were the specific remedy for violent delirium. The quantity of blood taken should be moderate; it should not often exceed sixteen or eighteen ounces at one operation: in a great many instances the loss of ten, twelve, or fourteen will be sufficient to effect the purpose, especially when combined with local applications, calculated to assist in bringing about the same result." 301.

The auxiliary means are obvious enough, as shaving the head—cold lotions—leeches or cupping—blisters. Some discrimination ought to be made when these last are employed. Dr. Prichard properly observes that, when the head is hot, and the face flushed, blisters on the scalp itself are injurious; it is when the external parts of the head are cold, while symptoms exist which indicate a determination to the deep-seated encephalic vessels, that blisters may be beneficial. "General bleeding will scarcely be required in two cases out of ten of enteric mania." The treatment directed towards the alimentary canal must be the same as that described for epilepsy. Emetics may be useful, where there is no strongly marked fulness and increased action in the carotids and temporals, or after relief of this symptom has been obtained by bleeding. In many instances, Dr. P. observes,

---

* See page 143 *et seq.* of this volume.

a dose of calomel, with one grain of tartarized antimony, (as observed in page 144 of this volume,) will be sufficient. Sometimes, however, eight grains of the latter medicine will hardly be capable of overcoming the torpor of the stomach.

"The bowels ought to be completely purged by powerful cathartic doses, given as frequently as the strength of the patient will admit, until the full effect takes place. If they are slow in their operation, they should be assisted by purgative enemas, containing Oil of Turpentine with Castor Oil. We must not be withheld from the use of purgatives by an assurance that the bowels have been open, or even loose, for some time: even in this case they are often found to contain a great mass of fœculent matter." 303.

The disorder of this part of the system is of the same description as in enteric epilepsy, and requires the same treatment. There is no medicine, Dr. Prichard thinks, upon the whole, so valuable in these affections, as the rectified oil of turpentine. "It possesses a particular property of allaying irritation in the nervous system, at the same time that it restores a healthy action in the intestinal canal." When there is suspicion of worms, then this medicine is particularly indicated. The form of exhibition is an emulsion, as stated in our former article, from half a drachm to a drachm, (of the oil,) being taken three or four times in the day. If the medicine be given in large doses it will be likely to occasion hœmaturia—even in smaller quantities, it sometimes give rise to nausea and vertigo. The emulsion is less offensive to the stomach, than the oil in any other form. The diet should not be too low in enteric mania, as the disease is generally accompanied by, or gives rise to, great emaciation and debility. After the removal of the intestinal disorder, country air and exercise, with the use of the shower bath, will tend greatly to restore the health. It must be observed, however, that, in some cases, the mania will continue after the digestive organs are completely healthy in function. It is evident, that the symptomatic disorder of the brain has then become idiopathic —that is, a lesion of structure. We shall now present some account of a few of the cases related by Dr. Prichard.

*Case.* James Nott, a strong muscular man, aged 46, was admitted with mania on the 13th November, 1820, in a frantic state—his tongue furred—mouth and fauces beset with frothy mucus—pupils contracted—face flushed—eyes wild and glistening—pulse slow and full—pain on pressure in the region of the liver. He could give no account of himself, but his wife had observed that he was not well for some days before the attack, being costive, disinclined for food, restless, with head-ache, giddiness, and sleeplessness. After these symptoms had continued four or five days, he suddenly jumped out of bed one night, began to talk incoherently, and break the tables and chairs. This was about a fortnight before his admission. He had been in the habit of drinking freely formerly, but had led a sober life for the last two years. When his bowels were opened after the constipation, his wife remarked that the evacuations were very offensive, and looked like rotten flesh.

At the time of admission his face was flushed—his eyes wild and glistening—he was talking with much energy—asserting himself to be a man of great fortune—and declaring he was not mad. He was bled from the arm and the temporal artery—head shaved—leeches to the head and a blister to the neck—was purged—but all without diminishing the violence of the maniacal excitement. Three grains of emetic tartar produced no effect on his stomach—and a repetition of six grains only made him vomit occasionally. He was constantly restless, and obliged to be kept in a straight waistcoat. For five or six days after admission he took little or no food—his sleep was disturbed with raving fits—and he passed his evacuations involuntarily in bed. After the tenth day, some stimulants and opiates were tried, but soon discontinued. From that time he gradually sunk in a state of stupor, and expired on the 6th December, about five weeks from the commencement of the attack. During the whole of his illness there was a peculiar and strongly fetid smell emanating from him, and which was very perceptible in the body previously to dissection.

### *Post-Mortem Appearances.*

"*Abdomen.*—The intestines were distended with flatus, and also contained a considerable quantity of fæces: they were, in many places, more vascular than usual. There was no perceptible mark of inflammation in the external surface of the stomach; but, when slit open, its inner membrane was found to be considerably reddened: this appearance was more strongly marked about the cardiac portion: the inner coat of the duodenum was also more vascular than natural; and this intestine, as well as the stomach, contained much tenacious mucus, which adhered to the coats, and was not easily wiped off. The remainder of the canal was not examined. The liver was firm, but appeared healthy: the gall bladder contracted, and contained but little bile.

"In the thorax the right lung firmly adhered to the diaphragm, and the same part contained abscesses; one of which had formed an opening into the right thoracic cavity. This cavity was quite full of purulent matter, mixed with serum. The lung on the same side was quite collapsed, and the pleura costalis, and pleura pulmonalis, coated with a layer of coagulable lymph. The left lung adhered extensively to the side, but was otherwise healthy.

" *Head.*—Much fluid blood flowed when the longitudinal sinus was cut: the arachnoid membrane was much thickened, and almost opaque: there was effused fluid under it. The brain was firm: all the ventricles were full of serum. There was also serum at the basis of the brain." 308.

There is something not quite satisfactory in the report of this dissection. We do not understand how the right lung could be *firmly adherent* to the diaphragm, and "*quite collapsed*" at the same time. Be that as it may, the case affords a striking example of the extent to which disorganization will sometimes go in the chest without corresponding lesion in the function of respiration. We hear of no cough, no difficulty of breathing in this patient, and yet abscesses existed in the lungs, and one side of the chest was full of sero-purulent matter. It also offers an illustration of a remarkable pathological fact—namely, how a severe affection of the brain or its coverings will mask even a destructive process going forward in another part of the system. As far as the head was concerned, we consider the case as exhibiting an excellent specimen of arachnitis.

The second case related in this section by Dr. Prichard, is one of enteric mania pretty strongly marked. It was a youth, of 17 years of age, who had three temporary attacks of insanity, and each in the autumnal season of the year. The last attack was in October 1820, when he started from bed one morning (like thousands of others at that time, who were little less insane than himself) to go and meet the Queen! During this paroxysm he presented the following phenomena, viz. distention of the abdomen—pains in the abdomen and chest—voracious appetite—titillation of the nostrils—restlessness at night—discharge of worms in his motions. He was bled—had calomel and other purges, and then took the oil of turpentine in half-drachm doses thrice a day. This purged him violently, the stools containing a considerable quantity of blood. The tumour of the abdomen subsided, and he lost all symptoms of mania. Still he complained of pains in the head, and the carotids pulsated too strongly. He was cupped and blistered. He was discharged cured, and has had no relapse.

Passing over the third case, which at one time was hopeless, but was apparently saved by bleeding, we come to a poor black, a native of Jamaica, who being discharged from His Majesty's service in the Mediterranean, fell into want and distress at Malta, which terminated in mania. The predominant idea in his hallucination was, that he held converse with the Almighty, who often said to him—"never fear massa; me do great tings for you."

"From the date of his admission to the period of his death, the following were the symptoms under which he laboured, in respect to the natural and vital functions:—

"Pyrexia, dyspnœa, hurried circulation. Tension of abdomen, morbidly dry skin, voracious appetite, constipated bowels. After an uncertain period the above symptoms would be changed for the following:—Languor and debility; lowness of spirits; no appetite; tongue more furred than usual; pulse less frequent; effusion in the abdomen; diarrhœa. During this latter state of the case his hallucinations disappeared.

"He complained of pains, which he referred generally to the umbilicus and right hip-joint. These were partially relieved by remedies employed.

"On April 6, 1819, he was seized with enteritis, and died on the morning of the 8th." 313.

*Dissection.* There was great vascularity in the brain and its coverings—the cerebral mass itself very firm—ventricles distended with fluid. In the abdomen the viscera were all so agglutinated by adhesive inflammation as not to be separable without tearing the parts. The omentum was enlarged, firmly attached to the parietes of the abdomen, and when cut into, resembled pancreas. The surface of the intestines was covered with numerous small cartilaginous bodies. The mesenteric glands were as hard as cartilage.

We cannot but suppose that the visceral disorder here preceded and caused the cerebral and intellectual derangement.

The fifth case stated by our author is very satisfactory in shewing the diminution of the mental disorder, *pari passu* with the improvement of the natural functions. The patient was a young unmarried woman, who had been slightly affected with insanity about three months, but gradually getting worse. On admission she was constantly talking, laughing, or crying—bowels generally in a constipated state—tongue covered with a dark brown fur—breath very offensive—pupils more than naturally dilated. The head was shaved and blistered—she was briskly purged with calomel, jalap, and tartarized antimony—and ordered to use the shower bath every second morning. She was bled once, having a cough and oppression at the chest. Under this treatment the state of the chylopoietic functions improved, and the mental derangement disappeared. For several other interesting cases we must refer to the work itself.

*Hepatic Mania.* We now come to "maniacal affections connected with disease of the liver and other hypochondriac viscera." In all ages there has been a conviction among physicians of the relation between morbid states of the epigastric viscera and certain disorders of the mind, particularly dejection of spirits—hence the term hypochondriasis attached to habitual melancholy bordering on insanity. Dr. Prichard is disposed to view these affections of the hypochondriac viscera, as concomitants rather than causes of the desponding condition of the mind alluded to. In this we decidedly disagree with our author—for had we no other evidence than that of our own personal feelings, we could not entertain a doubt of these depressions of spirits, and irritability of temper, being frequently, nay generally, *caused* by derangements of function (whether the structure be altered or not) of the abdominal organs. Dr. Prichard observes that "there is, however, a much more firmly established malady sometimes existing in the viscera of the abdomen, in persons labouring under maniacal disorders." This malady was first pointed out conspicuously by Dr. Cheyne, in his work on comatose diseases, where he cites a statement of Mr. Todd, one of the surgeons to the House of Industry, "which, if strictly correct, is most remarkable," namely, that—"in every dissection he (Mr. Todd) has made after idiotism and mental derangement (*and he has made upwards of four hundred)* he has found the *liver* more or less diseased. He observes, after insanity, generally no great change of colour; but the organ is more bulky, with a thicker edge, and always connected by preternatural adhesions, sometimes of great extent, to the peritoneum." We have made enquiries respecting this remarkable statement, and we consider it our duty to say that there is an error in it. We absolve Mr. Todd from all wilful misrepresentation; but still (and we have it from the very best authority) the statement is incorrect. It would indeed be almost a miracle, if, out of four hundred dissections of maniacs, not one were found free from derangement of structure in the biliary organ. In Dr. Prichard's own experience the instances have not been very numerous in which organic disease of the liver, or other large viscera, has been discovered in conjunction with maniacal disorders. He confesses, however, that in the investigation of this subject he laboured under "some disadvantages," the nature of which he has not thought proper to state. Dr. Prichard subjoins the minutes but of one case of this form of disease—it is, however, a striking one.

A man, aged 42 years, tall and muscular, some of whose parental relations had been afflicted with madness, was admitted under a frenzy warrant. The corporeal symptoms, on admission, were, pain or sense of weight across the forehead, over the eyes—contracted pupils—white tongue—costive bowels—scanty and high-coloured urine—great restlessness. Depletion gradually restored him to apparent health of body and mind, and he was discharged in somewhat more than three months from his entrance into the hospital. A domestic irritation immediately lighted up the disorder of the mind anew, and he was obliged to be once more confined. By laxative medicines and the shower bath, the symptoms of mental disorder were subdued, but the patient was found to labour under considerable disease of the abdominal viscera. The abdomen was swelled, and painful on pressure—urine scanty—thirst troublesome—bowels irregular—cough, with oppression of breathing—chilliness. Purgatives, diuretics, and bitter aperients gave occasional relief, but he continued ailing for better than two years from the time of his re-admission, with very little remains, however, of his mental derangement, when, in October 1817, he was attacked with the prevailing typhus fever, and died suddenly on the second day of the new disease, without any previous indication of danger.

*Dissection.* Dura mater extensively and firmly adherent to the skull—and in several places, exhibiting indices of local inflammation—the vessels of the dura mater minutely injected—the membrane itself thickened, and evincing marks of chronic disease—some serous effusion between the dura and pia mater—the latter membrane diseased—on the surface and between the convolutions of the brain there was a layer of coagulable lymph—vessels of the cerebrum turgid, and the substance firm—considerable quantity of serous fluid in the ventricles. In the pericardium, was a good deal of serous fluid, and the heart bore marks of inflammation. In the abdomen, the liver was found generally enlarged, and its right lobe in a diseased state. The spleen was diseased; its substance like grumous blood. The kidneys were enlarged—the alimentary canal healthy.

Dr. Prichard thinks, that the primary seat of disease was in the liver, and that the encephalic disease was secondary, or symptomatic of the hepatic affection. We believe with Dr. Prichard, that abdominal obstructions have a stronger tendency to derange the cerebral functions and structure than *e converso;* but it is often very difficult to say in which class of organs the disease commences.

The ninth chapter of Dr. Prichard's work is on cerebral disease, giving rise to mania, and occasioned by the direct operation of noxious agents on the brain and nervous system.

He divides this chapter, or rather the cases composing it, (for it is principally occupied with cases,) into three parts:—according as the disorders result from mechanical injuries, physical causes, or mental emotions.

The instances are very numerous of mania following injuries of the head; and the rationale of such causes is so obvious, that we may pass over the subject entirely.

In respect to the class of physical causes acting directly on the brain or nervous system, Dr. Prichard notices them under distinct heads; viz. 1st. Chronic inflammation and its effects, as tumours, or other spontaneous changes of structure, often connected with scrofulous diathesis. 2d. Noxious matters taken into the stomach with the aliment or otherwise. Of the poisons which stimulate the nervous system and induce diseased action, some are medicines, for instance mercury, which, in some peculiar constitutions, induces maniacal affections. But the most frequent cause of insanity, under this head, is the immoderate use of wine or spirits. Dram-drinking is a very common precursor of madness among the lower orders of society. Where the predisposition to insanity, indeed, is very strong, diet alone, when too stimulating in proportion to the excitability of the constitution, may, and does bring out mental derangement. Patients of this class enjoy good health as long as they live abstemiously and take sufficient exercise, but become victims of this or some other disease, as soon as they indulge in full diet. External heat is not unfrequently the exciting cause of insanity—but especially the alternations of heat and cold. In this way we may often account for those periodical attacks of mania which occur every spring or summer in some people, unless they adopt certain means of obviating the effects of returning heat, and sudden vicissitudes of temperature. The cases related by our author, in the way of illustration, are sufficiently in point, and deserve the attentive perusal of the reader. The general line of treatment, in these cases, is touched upon in our last number, page 147.

The influence of moral causes, or rather of the mental emotions excited by them, in producing maniacal affections, is so notorious, that many physicians and others imagine the whole theory of such diseases to hinge upon this species of agency.

"The fact is, however, that the mental impressions and emotions of the lunatic are much more frequently the consequences, than the causes of his malady. Every insane person has some false impressions, and his hallucinations engender more or less of passion, and what seems to be morbid emotion. A lunatic, whose disorder had its original source in the state of the natural functions, and who would have continued sane during his life, if his bowels or liver had performed their proper actions, or if he had not induced a morbid excitement by dram-drinking, will talk wildly and vehemently upon whatever subject the current of his thoughts, and the habits of his former life, have rendered him most disposed to dwell upon. He contemplates them erroneously, mistaking some day dream for a reality; and the emotion which this erroneous impression excites, is supposed by those around him to be the cause of his disordered state: whereas the whole phenomena of his derangement are consequent upon a disease of the brain, induced by merely physical causes." 369.

Although this consideration may lead us to reduce very much, the number of maniacal cases hitherto referred to moral causes, still the existence of such a class cannot be questioned. It is nearly certain, that some organic operation of the brain is the physical cause, or at least, the universal antecedent of perception. It is, therefore, reasonable enough to suppose that, too strong and too vivid impressions of a moral nature may, and frequently do, derange the functions, or even the structure of the brain, and thus lead to insanity. A concomitant circumstance of strong mental emotion and of vivid thought, is almost invariably an increase of vascular action, or determination of blood to the brain. Of all kinds of mental emotion, there is none which operates so frequently in producing disease, as fear, or apprehension concerning the future. The propensity to look forward with hope and fear seems to be so necessary to self-preservation, that it is common to many, ("perhaps, says Dr. P. to *all*,") animated creatures. In the habits of the ant, the bee, and of birds, we especially observe the effects of this principle; but we greatly suspect that, in animals, it is merely an impulse of instinct, whereas, in man, it is almost entirely the offspring of reason, or a contemplation of the possibilities or probabilities of what may happen in future. In man too, there is no limit to this anxiety about the time to come.

"Even death, which the experience of our senses presents to every one as the, 'ultima linea rerum,' has never been with the generality of men, in any age or country, the boundary of their anxious expectations. Philosophers have endeavoured in vain to make them wiser in this respect. The propensity is universal and unalterable by circumstances; and it may hence be concluded, like the pursuit of pleasure, and the aversion to pain, to be a principle in the natural constitution of man.

"The dread of future evils, which in the present scene of existence is held in some restraint by the experience of realities, expands into infinitude after the barrier is passed. A thousand horrible forms hang over the path which the human imagination has marked out to its view through the regions of futurity. Hence death becomes the king of terrors: not by the simple horror of non-existence, but as ushering us into an unfathomable abyss of vague anxieties" 372

It is curious that, whereas our anticipations respecting the things of *this world* are usually of a cheerful nature, consisting of hopes rather than fears, yet the expectations which relate to the *future*, or unknown world, are generally of a gloomy and melancholy description, consisting of fears rather than hopes. Yet there seems to be no obvious cause why the ancient mythologists should have held out more of penal sufferings than agreeable prospects in the world of spirits. Our author thinks, and not without shew of reason, that the superstitions of mankind have not been merely the creations of the fancy, but principally of the *conscience*. It would seem, indeed, that a certain persuasion of moral demerit or delinquency has been a universal impression on the human mind, in all ages. With this, has been naturally associated the idea, that we are accountable beings, and that there are certain unseen powers, before whose tribunal we may, and probably will be, arraigned. Even philosophers have not always succeeded in emancipating themselves entirely from these apprehensions:—witness Voltaire, who, in his last illness, sent for a Capuchin, confessed, and received absolution.* But we are now verging on the grounds of the moralist or theologian; and, we are only concerned with this subject as far as the theory of religious insanity is connected with it. Now, although there would appear to be some innate propensity in the human mind to ponder on futurity, with some gloomy apprehensions respecting the state of departed souls, yet there can be no doubt that the frequency of insanity is considerably increased by the ideas which are publicly inculcated, (and, also, the *manner* of inculcating them,) by certain sectaries in religion. We are not sufficiently acquainted with the domestic history of the ancients to determine in what degree insanity prevailed among them; but we may reasonably suppose that, the superstitions which could induce mothers to put their infants into the mouths of crocodiles, must have abounded with sources of intense emotion, and frequently led to insanity.

"Even the classical faith of Agamemnon, who cut the throat of his daughter, in order to procure a change of the wind, must have produced many a religious lunatic besides Orestes. It would, perhaps, be rash to assert, and yet it can hardly be doubted, if human nature is every where alike influenced by similar causes, that the victims of superstitious terror were more numerous among the heathens of antiquity than they have been among the generality of Christian nations." 376.

---

* Grimm's Correspondence.

Although it is manifest that the pure doctrines of Christianity are calculated to tranquillize rather than disturb the mind, yet it must be confessed that, the doctrines of some churches and sects, are more favourable to the development of insanity than those of others. In the Romish church, the facility of obtaining absolution, and the doctrine of purgatory, are well calculated to appease the apprehensions of an alarmed conscience. On the other hand, the doctrine of predestination cannot fail to excite terror and dismay in the minds of those who are impressed with such a persuasion. Still, as Dr. Prichard well observes, much less depends on the creed or tenet, than on the style or mode of representation prevalent in any particular time or country. At present, in most of the catholic countries, (excepting France, of course, where there is no religion,) there is very little energy or vehemence of declamation, and facts would shew, Dr. P. affirms, that religious madness, is now comparatively rare among the inhabitants. In those countries, also, where the reformed religion has been long established, as in Scotland and the Dutch provinces, the clergy have laid aside the impassioned oratory for which they were once so remarkable—consequently they are nearly on a par with the catholic countries in respect to immunity from mania religiosa.*

"After all, our ideas of pathology will be very erroneous, if we do not bear in mind that a certain predisposition in the physical state of the brain is a necessary condition, in order that the above mentioned cause, however powerful as a moral agent, may give rise to insanity. Superstition is indeed itself a disease, but it is a disease of the mind: it is only when its influence is exerted on a nervous system, weak by organization, that the disorder in the corporeal organs, which constitutes insanity, is produced. We have ample proofs that the most abject and fearful superstition may exist, and may torment its victim through life, without giving rise to any insane hallucination, properly so termed." 379.

When fear and anxiety, in persons of maniacal predisposition, respect only the bodily health and sensations, and amount to nothing more than an unnecessary apprehension of diseases, accompanied by low spirits and extreme irritability, it is termed hypochondriasis; but when the hallucination arises to a certain pitch, as, for instance, when the patient fancies himself a tea-pot, it is manifestly insanity. "Anxieties of this sort are the consequences, not the causes, of a diseased action in the brain." This may be so; but we be-

---

* For many curious facts collected by Dr. Burrows in his able "Inquiry," we refer our readers to that publication.

lieve also, that this *diseased action* in the brain, is itself often a *consequence* of disordered function or structure in other, and distant parts of the body.

In a country like this, where commercial enterprize engages so many individuals in hazardous pursuits, grief, and especially disappointment, form frequent moral causes of insanity. It is well known that, during the horrors of the French revolution, the hospitals were filled with insane inmates, partly from the influence of grief and domestic calamities—partly through the violent passions excited in the struggle of political parties. In all these, and other cases of mania, arising from the effect of emotions, "there is considerable disorder of the natural functions." No doubt of it—we can have no idea of a moral cause producing insanity without first deranging some important function of the body. In this point of view, we can very easily believe the following assertion.

"I have repeatedly seen maniacal affections, which seemed to have been brought on by passions, relieved in a very short time by remedies which acted merely on the body." 381.

The moral *treatment* of the insane is now well understood by all—indeed, there is nothing in it which might not have been suggested by common sense and discretion. The cruel methods which have been adopted in some establishments, to the disgrace of our nation, must have owed their origin to carelessness and indifference, rather than mistaken ideas, since it is hardly possible to suppose that any medical practitioner could be so stupid as to expect, that any "beneficial effect could result from inflicting corporal severities in the cure of a disease of the brain." This is true; but yet, we occasionally meet with certain almost indomitable spirits that require measures apparently harsh, else the mental violence cannot be subdued, however we may act on the body by medicines. At the same time, we agree with our author in the following remark.

"I believe that most of the vauntings which are set forth, in the present time, respecting the greater skill displayed in certain establishments in the moral treatment of lunatics, and the greater humanity of certain practitioners, has its origin in motives which are easily traced, and which are unfortunately but too common in their operation." P. 382.

Our analysis has now extended so far, that we must pass over the subject of somnabulism or ecstasis, on which our author, as usual, makes many sensible observations, and ingenious remarks.

Dr. Prichard's work has afforded us much gratification and information on a most important class of human afflictionons. It stamps the author as a man of accurate observation, unwearied attention, and profound thought. Let him remember that his talents are lent to him, not merely as an annuity for himself, but also as a legacy for the public. Let him, therefore, persevere in improving those talents to the utmost of his power, and employing them for the benefit of mankind at large. His reward will be, an approving conconscience within, and a fairly earned reputation abroad. What can be more grateful to a sensible and virtuous mind?

